# The impact of stroke on emotional intelligence

**DOI:** 10.1186/1471-2377-10-103

**Published:** 2010-10-28

**Authors:** Michael Hoffmann, Lourdes Benes Cases, Bronwyn Hoffmann, Ren Chen

**Affiliations:** 1 Cognitive Neurology Division, Neurology Department, James A Haley VA Hospital, 13000 Bruce B. Down's Blvd, Tampa, Florida, 33612, USA; 2 Professional Center, Calle Munoz Rivera, Oficina 211, Caguas, 00725, Puerto Rico; 3 Management & Executive Education at the Crummer Graduate School of Business at Rollins College. 1000 Holt Avenue 2712, Winter Park Orlando, Florida, USA; 4 College of Medicine Biostatistics Core, University of South Florida, 12901 Bruce B. Down's Blvd, Tampa, Florida, 33612, USA

## Abstract

**Background:**

Emotional intelligence (EI) is important for personal, social and career success and has been linked to the frontal anterior cingulate, insula and amygdala regions.

**Aim:**

To ascertain which stroke lesion sites impair emotional intelligence and relation to current frontal assessment measurements.

**Methods:**

One hundred consecutive, non aphasic, independently functioning patients post stroke were evaluated with the Bar-On emotional intelligence test, "known as the Emotional Quotient Inventory (EQ-i)" and frontal tests that included the Wisconsin Card Sorting Test (WCST) and Frontal Systems Behavioral Inventory (FRSBE) for correlational validity. The results of a screening, bedside frontal network syndrome test (FNS) and NIHSS to document neurological deficit were also recorded. Lesion location was determined by the Cerefy digital, coxial brain atlas.

**Results:**

After exclusions (n = 8), patients tested (n = 92, mean age 50.1, CI: 52.9, 47.3 years) revealed that EQ-i scores were correlated (negatively) with all FRSBE T sub-scores (apathy, disinhibition, executive, total), with self-reported scores correlating better than family reported scores. Regression analysis revealed age and FRSBE total scores as the most influential variables. The WCST error percentage T score did not correlate with the EQ-i scores. Based on ANOVA, there were significant differences among the lesion sites with the lowest mean EQ-i scores associated with temporal (71.5) and frontal (87.3) lesions followed by subtentorial (91.7), subcortical gray (92.6) and white (95.2) matter, and the highest scores associated with parieto-occipital lesions (113.1).

**Conclusions:**

1) Stroke impairs EI and is associated with apathy, disinhibition and executive functioning. 2) EI is associated with frontal, temporal, subcortical and subtentorial stroke syndromes.

## Background

Emotional intelligence (EI) is a concept that may be defined in different ways by the psychological and medical disciplines that are concerned with its importance. The four-branch model (perceiving emotions, facilitating thought, understanding emotions, managing emotions) of emotional intelligence definition by Mayer and Salovey is a concept that appears popular [1]. Additionally, Bar-On has conceptualized the EI construct as comprising the ability to (i) understand emotions and express feelings, (ii) understand how others feel and relate with them (iii) manage and control emotions, (iv) use emotions in adapting to one's environment and (v) generate and use positive affect to be self-motivated in coping with daily demands, challenges and pressures [2]. EI is important for personal, social and career success [3]. EI has been studied in both healthy people and after brain illness. For example studies of specific healthy populations including nurses and doctors have also determined that high EI results in improved patient relationships and outcomes. Suboptimal physician patient communication has been correlated with increased risk of patient complaints and malpractice claims in a Canadian study of patient physician communication scores [4-6]. Studies of the most common cerebral disorders, namely stroke and dementia are beginning to implicate dissolution of the components of emotional intelligence. Frontotemporal lobe disorders (FTLD), the most common cause of dementia under the age of 60, present with frontal and behavioral symptoms and syndromes, including disorders of emotion, empathy violation of social and moral norms [7]. Furthermore, stroke, Alzheimer's disease (AD) and FTLD are regarded as a continuum of disorders in a clinical phenotypic, pathologic and genotypic sense [8], with overlap syndromes common and the need for even more precise clinical acumen to differentiate these disorders.

EI has been linked to the frontal anterior cingulate, insula and amygdala regions [9]. EI and stroke have rarely been formally investigated with only 2 references found by Pubmed search [10,11] in addition to other brain lesion models [12]. The overall approach for holistic brain injury assessment should be neurological, neuropsychiatric, cognitive, behavioral and emotional. Only neurological deficit is recorded in current stroke assessment scales, yet the others may be the most important from a family, social, career and rehabilitative point of view. EI has been embraced by the corporate world because of its perceived translation into social and career success [13-15]. Importantly, it is amenable to cognitive and behavioral intervention programs [16,17].

## Aim

To ascertain which stroke lesion sites impair emotional intelligence and how this relates to contemporary frontal assessment measurements.

## Methods

Consecutive patients, aged 18-90 years were accrued through a prospectively coded, dedicated cognitive stroke registry, as part of a tertiary care primary JCAHO (Joint Commission on Accreditation of Healthcare Organizations) and comprehensive AHCA (Agency for Health Care Administration) Stroke Center from January 2003 to December 2006. All patients (n = 2389) were examined and managed by board certified neurologists. The cognitive bedside tests were administered by trained stroke team members comprised of residents and stroke research nurses who also graded stroke severity. This cognitive bedside test screened for a range of cognitive disorders in addition to the assessment of aphasia. The Stroke registry was approved by the University Institutional Review Board and in compliance with HIPAA regulations. All patients signed informed consent for the evaluation and the collection of the their neurological, medical and neurocognitive data. Non aphasic, independently functioning patients post stroke were evaluated with the Bar-On emotional intelligence test (EQ-i) which is a self report, Likert scale assessment, yielding an Emotional Quotient and is a standardized psychometric measure of various aspects of emotional and social intelligence [2]. The test was usually administered within the first month after stroke but with a range from 1 week to 6 months post stroke. In addition, frontal tests that purport to measure executive function and other cognitive domains were used. These included the Wisconsin Card Sorting Test (WCST) [18] and the Frontal Systems Behavioral Inventory (FRSBE) [19], for correlational validity. The FRSBE is a family and self rated, normed, scoring instrument that reports measures before and after illness for apathy, disinhibition, executive function as well as a total score. A screening, bedside frontal network syndrome test (FNS) [20] for initial cognitive evaluation and NIHSS [21] to document neurological deficit were also recorded. Lesion location was determined by the Cerefy digital coxial brain atlas [22].

### Exclusions

A history of dementia or other neurodegenerative disease, moderate or severe depression (because of its effect on cognitive testing), inability to complete all the subtests, substance abuse and less than 8 years educational level. Persistent obtundation, metabolic derangement, encephalopathy or coma was recorded but cognitive testing performed only in those recovering sufficiently within a month. Completion of both the screening and cognitive metric tests was necessary for inclusion in the series, which yielded 100 patients for analysis.

## Results

### 1. Demographics of patient study group (n = 100)

After exclusions, one hundred consecutive patients were eligible for analysis but because of missing data (n = 8), patients tested included (n = 92, mean age 50.1, CI: 52.9, 47.3 years), men n = 53 (58%), women n = 39 (42%) race ethnicity; Black (n = 10), Hispanic (n = 8), White (n = 72), other (n = 2). The mean education level in years was 13.8 years (95% CI: 14.4; 13.3, maximum 20 years and minimum 8 years). Overall, 38/92 (41%) of patients tested, irrespective of stroke lesion site, had abnormal EI scores as assessed by published normative data [2].

### 2. Correlational validity

EI total scores were negatively correlated with all FRSBE T sub-scores (apathy, disinhibition, executive, total) and the self-reported scores correlated better than family reported scores. The WCST error percentage T score did not correlate with the EI scores (Table 1). The screening frontal examination (FNS) correlated well with total EQ (0.408, p < 0.01).

**Table 1:** EQ Total and EQ sub-scores versus FRSBE scores

FRSBE	EQ Total score	EQ intrapersonal	EQ interpersonal	EQ stress management	EQ adaptability	EQ mood score
SA	-0.546**	-0.518**	-0.353*	-0.421**	-0.470**	-0.443**
SD	-0.454**	-0.330**	-0.313*	-0.469**	-0.477**	-0.219
SE	-0.579**	-0.492**	-0.333*	-0.486**	-0.614**	-0.390**
ST	-0.595**	-0.500**	-0.385*	-0.527**	-0.584**	-0.406**
FA	-0.342*	-0.270*	-0.229	-0.357**	-0.373**	-0.171
FD	-0.277*	-0.196	-0.049	-0.370**	-0.323**	-0.115
FE	-0.334**	-0.247*	-0.145	-0.408**	-0.396**	-0.103
FT	-0.386**	-0.310*	-0.21	-0428**	-0.402**	-0.177

Legend

S: Self report

F: Family report

A: Apathy

D: Disinhibition

E: Executive Function

T: Total score

* p-value at the 0.05 level of significance

** p-value at the 0.01 level of significance

### 3. Lesion site

**Figure 1:**
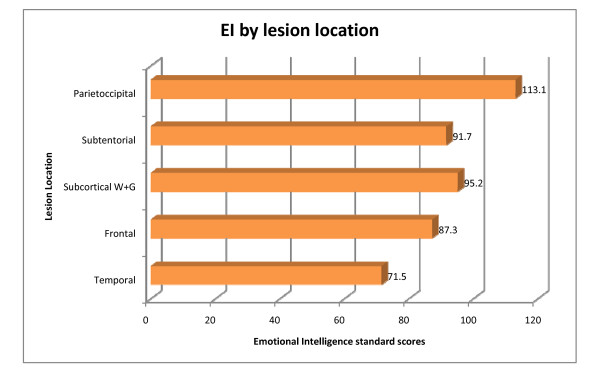
**Emotional intelligence scores according to stroke lesion site**.

The analysis of variance (ANOVA) test indicated that there were significant differences between the EI scores among the 6 lesion sites (F value 5.12, p = 0.0004). The lowest EI scores (reported in standard scores where 85-115 is within the normal range) were in the temporal lobe lesions (71.5), followed by the frontal lesions (87.3), subtentorial (91.7), subcortical gray matter lesions (92.6), subcortical white matter lesions (95.2) and parieto occipital lesions (113.1), (Figure 1). Of the 72 supratentorial lesion sites (subtentorial n = 20), the laterally of stroke included right (n = 37; 51%), left (n = 25; 35%) and bilateral (n = 10) lesions, (Table 2). In addition in 24 cases lesions were in 2 or more sites in the brain such as stroke lesions involving more than 1 lobe of the brain or both subcortical and cortical lesions. Not unexpectedly, in the subtentorial group (n = 20), because of the central anatomical vascular distribution of the basilar artery, lesions were almost equally distributed; right (n = 9), left (n = 8) and bilateral (n = 3).

**Table 2:** Stroke neuro anatomical supratentorial lesion sites.

Region of Interest	Right	Left	Bilateral	Total
Temporal	5	1	0	6
Frontal	17	10	5	32
Subcortical Gray	4	5	2	11
Subcortical White	4	7	2	13
Parieto occipital	7	2	1	10

**Total**				**72**

### 4. Five EI subcategory scores

Intrapersonal EI correlated with all the FRSBE scores except family reported disinhibition. Interpersonal EI correlated only with the FRSBE self reported scores and not family reported scores. The stress management and adaptability EI scores correlated with all the FRSBE scores. The EI general mood scores correlated only with the self reported apathy, executive and total scores (Table 1).

### 5. Stroke severity and EI scores

There was a weak relationship between stroke severity as measured by the NIHSS and EI scores (Pearson correlation -0.239 significant at the 0.05 level).

### 6. Regression analysis

Age and FRSBE total scores were significant influential variables to total EI. With 1 year of age increase, the total EI will increase 0.29 (p = 0.0144) and with 1 FRSBE self report T score increase, the total EI will decrease 0.63 (p < 0.0001). The regression equation; Total EI = 117.838 + .279 (Age) - .621 (FRSBE-S-T).

## Discussion

**Figure 2:**
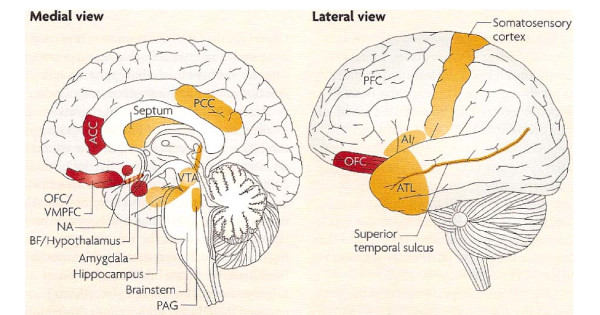
**Emotional Brain: Core (red colored) and Extended brain (orange colored) regions.** (Reproduced with permission Nature Publishing Group). Core Emotional brain. OFC: orbitofrontal cortex. VMPFC: ventromedial prefrontal cortex. ACC: anterior cingulate cortex. BF: basal forebrain. NA: nucleus accumbens. Extended Emotional Brain. PAG: periacqueductal gray matter. ATL: anterior temporal lobe. AI: anterior insula. PCC: posterior cingulate cortex. VTA: ventral tegmental area.

The main findings of this study concur with recent basic neuroscience postulates with respect to the widely distributed emotional circuitry in the brain as well as the close-knit emotion and cognitive processes. Perusal of figure 2 (with permission, Nature Publishing Group) of the more recently appreciated core and extended emotional regions of the brain does indeed represent a widely distributed cerebral network [9]. Our research with the "lesion method", agrees with this model in that diverse lesions within the stroke pathophysiological model were associated with lowered EI scores.

Clinical evidence implicating in particular the orbitofrontal cortex as part of the neural network for emotional intelligence has been suggested by previous researchers. Baron first reported emotional quotient impairment with orbitofrontal cortex lesions [10]. Likewise, in the study of Shamay-Tsoory et al, patients with prefrontal lesions, particularly with lesions of the orbitoprefrontal and medial frontal regions were significantly impaired in both cognitive and affective empathy as compared to parietal patients and healthy controls [11]. Furthermore, those with damage restricted to the prefrontal cortex, no matter which side, resulted in impaired empathy. Finally in their study, lesions involving the right hemisphere, patients with parietal lobe lesions were also impaired. We showed that a much more widely distributed lesion site network impairs EI, in keeping with the extensive contemporarily appreciated neurobiological emotional network. Many different brain lesions may affect EI and in our study, EI is associated with frontal, temporal, subcortical and even subtentorial stroke syndromes. However the strongest relationship at least by EI scores pertained to the frontal and temporal regions of the brain. This finding supports Pessoa's proposed extended emotional brain concept [9].

The neurobiological emotional network known as the Papez circuit, has been regarded as outdated and some have recommended that use of the term "limbic system" be abandoned [9,22]. The main reasons relate to the hippocampus, which is not part of the circuitry and the orbitofrontal cortex, which is part of it, but not included in the Papez circuit [23,24]. According to Pessoa, the emotional brain core components include the amygdala, nucleus accumbens, hypothalamus, orbitofrontal cortex, anterior cingulate cortex and ventromedial prefrontal cortex. Emotional brain extended areas include the brainstem, ventral tegmental area, hippocampus, periacqueductal gray matter, septum, basal forebrain, anterior insula, prefrontal cortex, anterior temporal lobe and posterior cingulate cortex [9].

There is evidence for a close interplay of cognitive and emotional brain circuits. The amygdala in particular, is viewed as the prime candidate for the emotion-cognition integration. The amygdala has a unique position at the geometric center of topological map and because of its extensive connections to other brain regions. Executive control is required for autonomy to override instinctive or prepotent responses with particularly important components in this network being the lateral prefrontal cortex (LPFC) for temporal information maintenance, the parietal cortex and PFC attention maintenance and the anterior cingulate cortex (ACC) for conflict detection and error monitoring. The orbito frontal cortex (OFC) and medical PFC are considered components in computing outcomes expectations. The neurochemical dimensions to these circuits include dopamine from VTA and SN (compacta) which projects to the nucleus accumbens (NA) and PFC for prediction and expectation of future rewards - a function of the dopaminergic system. Pessoa argues that one cannot separate cognitive and emotional brain contributions to executive control; "emotion and cognition conjointly and equally contribute to the control of thought and behavior. Each behavior has both affective and cognitive components, which have their biological basis in dynamic coalitions of networks" [9]. In our study, the emotion cognition interface was not specifically researched but the results of particular interest being that EI is correlated with executive function as well as apathy and disinhibition function scores.

Pathophysiological processes are important in our understanding of brain behavior relationships [25,26]. The stroke model is in a sense a "cleaner" more precise lesion method than neurodegenerative, traumatic, epilepsy or metabolic brain injuries. Cerebrovascular disorders frequently involve the frontal subcortical circuits involved in emotional and cognitive networks. Neither the commonly used stroke scales nor the bedside cognitive test, the Mini-Mental State Examination (MMSE) address these frontal network syndromes that may be the very first and most prominent manifestation of the disease. Neuropsychological tests including those focusing on frontal networks also do not capture the EI aspects at all. Specific EI tests such as the Bar-On [2] and MSCEIT (Mayer, Salovey, Caruso Emotional Intelligence Test) [27] are required to diagnose EI impairment although tests such as the FRSBE [19] and BRIEF [28] do provide some information about emotional disarray. These may be the most important deficits for people to realize, accommodate and treat.

Potential criticisms of the study relate to the methodology of testing EI and in the brain lesion determination. Self-report testing of EI as is done by the Bar-On EQ-i test as opposed to the MSCEIT remains an area of contention with some studies reporting a low correlation between two methodologies [29,30]. Brain lesions may be silent, old, incidental or undetected by standard multimodality MR imaging as is the case with diaschisis or neurochemical lesions without anatomical signature lesions (frontal hypometabolism with depression for example). Finally correlational analyses might be better performed with some of the newer composite frontal tests such as the DKEFS [31] or others focusing on specific areas such as the Iowa Gambling Test [32].

## Conclusions

Stroke impairs EI and is associated with the three principal frontal syndrome complexes of apathy, disinhibition and dysexecutive functioning. In addition it was demonstrated that an extensive emotional network, at least by lesion analysis, impairs EI. Does EI testing really matter? Neuroplasticity is an inherent process whereby the brain shapes itself through repeated experiences. The corresponding neural connections are strengthened and the ones less used, weakened [33]. The discovery of the relatively late maturation of the prefrontal circuitry for modulation of emotion suggests a neurological window of opportunity for helping children (or adults), for example to learn the best EI repertoire [34]. With the newly appreciated concept of adult neurogenesis and ongoing neuroplasticity, one may extrapolate that this applies to people with stroke or traumatic brain injury.

## Competing interests

The authors declare that they have no competing interests.

This includes;

1. Financial competing interests

In the past five years none have you received reimbursements, fees, funding, or salary from an organization that may in any way gain or lose financially from the publication of this manuscript, either now or in the future.

2. There is no organization financing this manuscript.

3. None hold any stocks or shares in an organization that may in any way gain or lose financially from the publication of this manuscript, either now or in the future.

4. None hold or are currently applying for any patents relating to the content of the manuscript.

5. None have received reimbursements, fees, funding, or salary from an organization that holds or has applied for patents relating to the content of the manuscript.

6. There are no other financial competing interests.

7. Non-financial competing interests

There are there no non-financial competing interests (political, personal, religious, academic, ideological, intellectual, commercial or any other) to declare in relation to this manuscript.

## Authors' contributions

MH conceived of the study methodology with assistance, collected the data, helped analyze and wrote the manuscript, RC performed all statistical analyses, BH contributed to the conception, design of study and critical review of intellectual content of data and LBC contributed to the acquisition, presentation of data and review of intellectual content of data.

All authors have read and approve of the content of this manuscript.

## Pre-publication history

The pre-publication history for this paper can be accessed here:

http://www.biomedcentral.com/1471-2377/10/103/prepub
